# Validation of the American Joint Commission on Cancer (8th edition) changes for patients with stage III gastric cancer: survival analysis of a large series from a Specialized Eastern Center

**DOI:** 10.1002/cam4.1118

**Published:** 2017-09-14

**Authors:** Jun Lu, Chao‐Hui Zheng, Long‐Long Cao, Shao‐Wei Ling, Ping Li, Jian‐Wei Xie, Jia‐Bin Wang, Jian‐Xian Lin, Qi‐Yue Chen, Mi Lin, Ru‐Hong Tu, Chang‐Ming Huang

**Affiliations:** ^1^ Department of Gastric Surgery Fujian Medical University Union Hospital Fuzhou China; ^2^ Department of General Surgery Fujian Medical University Union Hospital Fuzhou China; ^3^ Key Laboratory of Ministry of Education of Gastrointestinal Cancer Fujian Medical University Fuzhou China; ^4^ Fujian Key Laboratory of Tumor Microbiology Fujian Medical University Fuzhou China; ^5^ Department of Epidemiology and Health Statistics Fujian Medical University Fuzhou China

**Keywords:** gastric cancer, surgical oncology, survival, TNM stage

## Abstract

The 8th edition of the TNM was released in 2016 and included major revisions, especially for stage III. We aimed to compare the prognostic value of the 7th and 8th editions of the AJCC TNM classification for stage III gastric cancer. Clinical data from 1557 patients operated on for stage III gastric cancer according to the 7th edition between 2007 and 2014 were analyzed and compared using the 7th and 8th TNM classifications. A proposed staging system was established, and the three systems were compared in terms of prognostic performance. The stage shifted for 669 (42.96%) patients. It shifted from IIIA to IIIB (one patient, 0.06%), IIIB to IIIA (230 patients, 14.8%), IIIB to IIIC (94 patients, 6.0%), and IIIC to IIIB (344 patients, 22.1%). However, the new AJCC subgroupings did not prove distinctive for survival levels between T3N3aM0 (stage IIIB) and T3N3bM0 (stage IIIC) or between T4aN3aM0 (stage IIIB) and T4aN3bM0 (stage IIIC) when <30 lymph nodes (LNs) were resected. The performance of the 8th edition (c‐index, 0.614; 95% confidence interval [CI], 0.596–0.633) revealed no relevant improvement compared to the 7th edition (c‐index, 0.624; 95% CI, 0.605–0.643). The proposed staging system generated the best prognostic stratification. The 8th TNM edition may not provide better accuracy in predicting the prognosis of stage III gastric cancer. The proposed staging system, comprised of a combination of the number of LNs harvested and the 7th and 8th AJCC classifications, may improve predictive capacities for stage III gastric cancer.

## Introduction

The AJCC TNM system is recognized as the best malignant tumor staging system worldwide. Over recent decades, the AJCC TNM staging system has been revised continuously, and the most recent 8th edition of the TNM classification published in 2016 replaced the 7th edition from 2009 1. Changes to the latest classifications of gastric cancer were mainly based on data analyses from the US and Japan.

**Figure 1:**
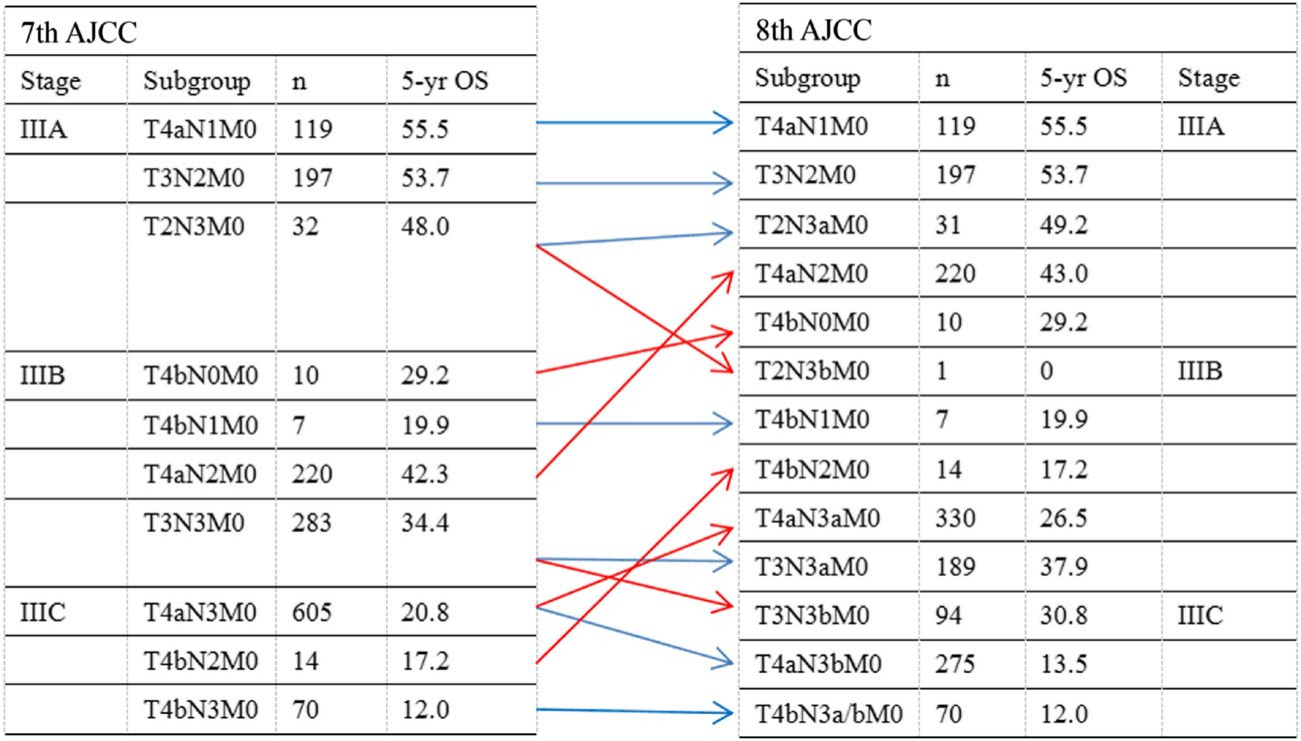
AJCC stage and TNM subgroup distributions of patients according to the 7th and 8th editions of the TNM calssification.

The 7th edition N3 stage was divided into N3a (7–15 positive regional lymph nodes, LNs) and N3b (>15 positive regional LNs). However, in the 7th edition, the N3 subclassification (N3a and N3b) was not incorporated into the final staging stratification. In other words, N3a and N3b do not differ with regards to the final pathologic stage 2. Recently, the AJCC published the 8th edition of the TNM classification, and several changes to the 8th edition of the AJCC staging system for gastric cancer have been proposed (Table S1). A key change adopted in the new edition details pN3 as pN3a and pN3b in the final pathologic stage. Thus, a comparison of stage distributions between old and new TNM classifications shows that stages I and II did not change except for T1N3bM0 (changing from IIB in the 7th edition to IIIB in the 8th edition). The main modification involved a major change to stage III. T2N3bM0 tumors were upstaged from stage IIIA to IIIB, and T3N3bM0 tumors were upstaged from IIIB to IIIC. In addition, T4bN0M0 and T4aN2M0 tumors were downstaged from IIIB to IIIA. Finally, T4aN3aM0 and T4bN2M0 tumors were downstaged from IIIC to IIIB (Fig. 1). As stated above, the most important change made to the 8th edition concerned stage III gastric cancer. Therefore, in this study, we mainly evaluated classification changes made in regards to stage III gastric cancer.

To the best of our knowledge, this study is the first study to examine GC classification using the 8th TNM system. The objective of this study was to evaluate the validity of the proposed 8th edition AJCC system and to identify the optimal TNM classification for stage III gastric cancer based on a prospectively collected database from a large specialized center in China.

## Patients and Methods

This study was designed as a retrospective analysis based on prospectively collected data. Between 2007 and 2014, 1557 patients (1148 men and 409 women representing a male‐to‐female ratio of 2.8:1.0; median age: 62 years; range: 17–101 years) underwent curative resection with D2 lymphadenectomy for stage III gastric cancer, according the 7th edition of the AJCC TNM classification 2, at the Department of Surgery at Fujian Medical University Union Hospital (Table 1). Histology confirmed an adenocarcinoma (ICD‐0‐3M‐8140/3, M‐8142/3 through M‐8145/3, M‐8210/3, M‐8211/3, M‐8255/3, M‐8260/3 through M‐8263/3, M‐8310/3, M‐8323/3, M‐8480/3, M‐8481/3, M‐8490/3) 3. The extent of D2 lymphadenectomy was defined according to Japanese Gastric Cancer Association criteria 4. The data from these patients included information on demographic parameters, information on operation techniques, histopathologic tumor characteristics, and survival rates. The lymph node ratio (LNR) was defined as the number of positive lymph nodes divided by the number of examined lymph nodes 5. We excluded the following patients from the study: (1) patients with pathological stage I or II conditions, (2) patients undergoing palliative surgery, (3) patients with distant metastasis, and (4) patients with synchronous malignancies. (5) We excluded 115 patients who had undergone neoadjuvant chemotherapy because, a special postneoadjuvant therapy stage (ypTNM) grouping system was provided in the 8th edition AJCC cancer staging manual.

**Table 1:** Clinicopathologic characteristics of the patient cohort

Characteristics	Total *N* = 1557	
	*N*	%
Sex		
Male	1148	73.7
Female	409	26.3
Age, years; median (range)	62 (17–101)	
Tumor location		
Upper	543	34.9
Mid	397	25.5
Lower	617	39.6
Tumor size, cm; median (range)	6.0 (1.0–20.1)	
Grade		
Low(1–2)	714	45.9
High(3–4)	843	54.1
Lymphatic vessel invasion		
No	1108	71.2
Yes	449	28.8
Vascular invasion		
No	1419	91.1
Yes	138	8.9
LNs resected; median (range)	32 (5–108)	
Lymph node ratio; mean (Standard deviation)	34.67 (24.59)	
pT category		
2	38	2.4
3	480	30.8
4a	935	60.1
4b	104	6.7
pN category		
N0	10	0.6
N1	129	8.3
N2	422	27.1
N3a	600	38.6
N3b	396	24.4

All operations were performed by experienced surgeons. Adjuvant chemotherapy with 5‐fluorouracil (5‐FU)‐based regimens (mostly 5‐FU with cisplatin) was recommended to the eligible patients. Among the 1557 patients, 81% (*n* = 1261) received adjuvant chemotherapy. Postoperatively, patients were examined during follow‐up visits every 3 months for the first 2 years and every 6 months thereafter. At each follow‐up visit, carcinoembryonic antigen, and carbohydrate antigen 19‐9 levels were measured. Thoracicoabdominal and pelvic computed tomographic scanning or abdominal ultrasonography was performed alternately every 3–6 months. Gastroscopy was performed yearly. In all, 1429 patients (91.8%) were followed up, and 8.2% (128/1557) of cases were lost to follow‐up. The median follow‐up duration was 51 months (range 1–113).

This study was approved by the Institutional Review Board of the Ethical Committee of Fujian Medical University Union Hospital.

### Definitions of the 8th edition TNM classification

For the 8th edition pTNM classification, definitions of T and N classifications were not changed, and only the final staging assignment of the pN3 classification was changed. The 7th edition pN3 classification was divided into pN3a and pN3b classifications in the 8th edition 1. Table S1 shows detailed classifications based on the 7th and 8th editions of the AJCC TNM classification.

### Statistical analysis

All data were analyzed with a statistical analysis program package (SPSS 17.0, SPSS Inc., Chicago, IL, X‐tile program, Version 3.1.2, Yale University, and the statistical software package, “R”, version 2.11.1, the R Foundation for statistical computing). Survival time was calculated from the day of surgical resection, and the day of death or last follow‐up was considered the endpoint. Overall survival (OS) was calculated using the Kaplan–Meier method, and the log‐rank test was employed to determine significance. The optimal cutoff points for the number of LNs harvested were calculated using the X‐tile program, which identified the cutoff with the minimum *P*‐values from log‐rank Chi‐square statistics for the categorical LNs in terms of survival 6. The predictive accuracy of the model was also evaluated by the concordance index (C‐index) 7, the corresponding confidence interval (CI) was obtained by bootstrapping, and a higher C‐index value represents more accurate stratification, as previously described 8. We used the likelihood ratio Chi‐square test, the linear trend Chi‐square test, and the Akaike information criterion (AIC) within the Cox regression model to compare the performance of the three staging systems. A higher likelihood ratio chi‐square score indicates better homogeneity, a higher linear trend chi‐square score indicates better discriminatory ability and monotonicity, and smaller AIC values represent better optimistic prognostic stratification 9. Two‐sided statistical tests were performed, and a *P* < 0.05 was considered statistically significant.

## Results

### Patient characteristics

Data for 1557 consecutive patients were analyzed. In total, 1148 (73.3%) of the patients were male, and 409 (26.3%) were female, with a median age of 62 years (range 17–101 years). The patient and histopathological characteristics are shown in Table 1.

### Identification of the cutoff number of LNs retrieved

**Figure 2:**
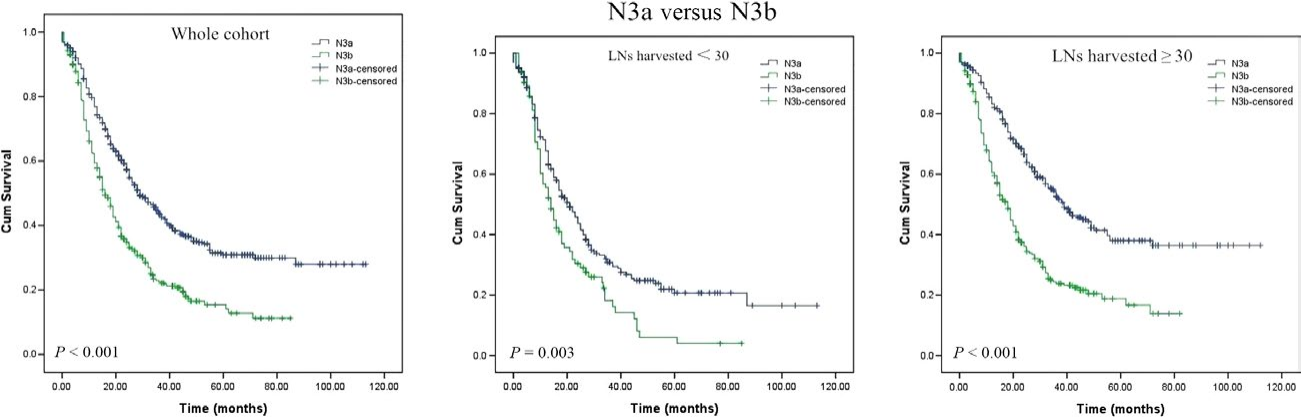
OS of N3a and N3b stage gastric cancer according to the number of LNs resected.

The X‐tile plots shown in Figure S1 illustrate that the highest chi‐square log‐rank value of 20.333 was achieved when 30 was applied as the cutoff value for LNs retrieved. Furthermore, the overall survival rates of stage N3a and N3b patients based on the number of LNs resected are shown in Figure 2. Patients with stage N3a diseases exhibited a significant survival benefit compared to patients with N3b diseases regardless of the number of LNs harvested.

### 7th and 8th editions of the AJCC TNM classification

Classification according to the two editions revealed that 996 patients with N3 tumors were divided into 600 patients with N3a (60.2%) and 396 patients with N3b (39.8%). The AJCC stage distributions according to the 7th and 8th editions of the TNM classification are shown in Figure 1. Our comparison of the two classifications revealed that AJCC III stage tumors changed in 669 patients (42.96%). In detail, patients were reclassified from AJCC stage IIIA to IIIB (one patient, 0.06%), IIIB to IIIA (230 patients, 14.8%), IIIB to IIIC (94 patients, 6.0%), and IIIC to IIIB (344 patients, 22.1%).

### Survival

**Figure 3:**
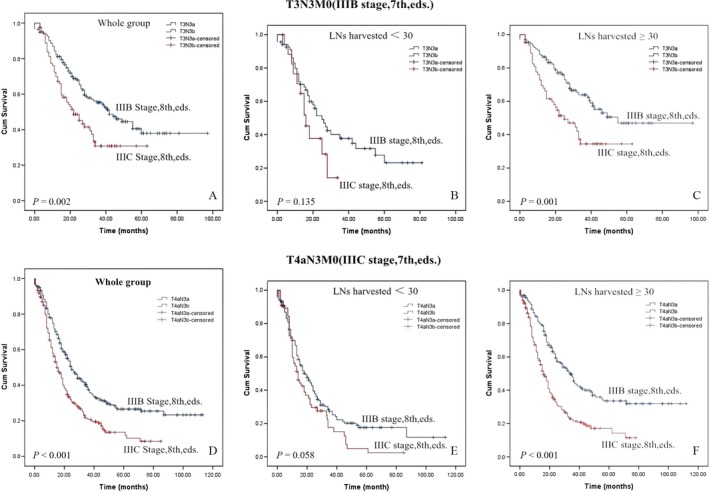
Comparsion of survival curves for patients with AJCC tumor stage shift.

Five‐year OS rates for patients whose TNM categories or AJCC stages changed between the 7th and the 8th editions are shown in Figure 1. All T3N3M0 tumors were categorized as N3a or N3b (Fig. 3A), and T3N3M0 tumors were subdivided according to the number of lymph nodes harvested (Fig. 3B and C). All T4aN3M0 tumors were categorized as N3a or N3b (Fig. 3D), and T4aN3M0 tumors were subdivided by the number of lymph node harvested (Fig. 3E and F). The 8th edition's division of T3/4aN3M0 tumors into T3/4aN3aM0 and T3/4aN3bM0 tumors reveals a statistically significant difference when ≥30 lymph nodes were harvested (Fig. 3C and F). However, the division had no prognostic impact when <30 lymph nodes were harvested in our series (Fig. 3B and E).

**Figure 4:**
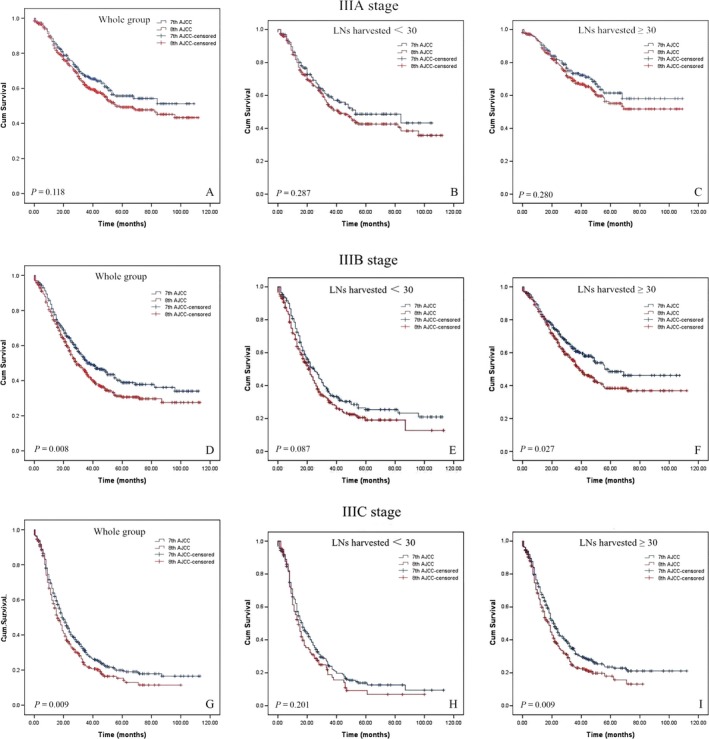
Five‐year survival probabilities for IIIA, IIIB, and IIIC stage.

Five‐year OS probabilities for IIIA, IIIB, and IIIC were 51.6%, 33.6%, and 18.2% for both groups and 55.7% versus 49.2%, 37.7% versus 30.1%, and 19.9% versus 15.3% for the 7th edition AJCC versus the 8th edition AJCC, respectively (Fig. 4A, D and G). Differences in cumulative survival rates for the 7th and 8th edition TNM classifications were not significant when the number of resected LNs was <30 (Fig. 4B, E and H).

### Comparisons among the three prognostic classification systems

**Figure 5:**
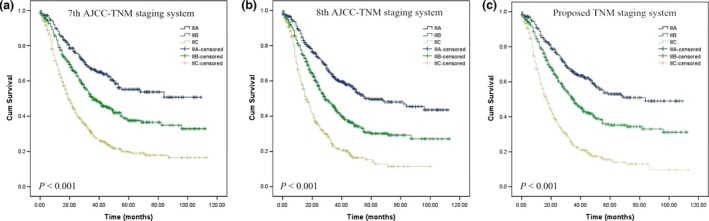
Comparsion of survival curves according to the seventh (A), eighth (B) American Joint Committee on Cancer TNM systems, and proposed (C) TNM staging systems.

We propose a novel staging system that combines the number of harvested LNs with the 7th and 8th edition AJCC TNM classifications as shown in Table 2. Cumulative survival rates according to the 7th edition, 8th edition and proposed TNM stage classifications are shown in Figure 5A–C. The performance of the 7th edition, 8th edition, and proposed staging system assessed by the C‐index, AIC, likelihood ratio chi‐square score, and linear trend chi‐square score are presented in Table 3. A statistical assessment of the prognostic performance of the 2 AJCC classification editions based on the c‐index reveals a value of 0.624 (95% CI, 0.605–0.643) for the 7th edition and a value of 0.614 (95% CI, 0.596–0.633) for the 8th edition. We found no improvement in the prediction of patients’ prognoses for the 8th edition. With a value of 0.646 (95% CI, 0.628–0.665), the c‐index of the “proposed staging” exhibits a higher level of prediction efficiency than both AJCC classifications (Table 3). Compared to the 7th and 8th editions of the system, the revised system shows more gradient homogeneity (higher likelihood ratio chi‐square score) and monotonicity (higher linear trend chi‐square score). Furthermore, the proposed system generated a smaller AIC value, denoting optimum prognostic stratification.

**Table 2:**
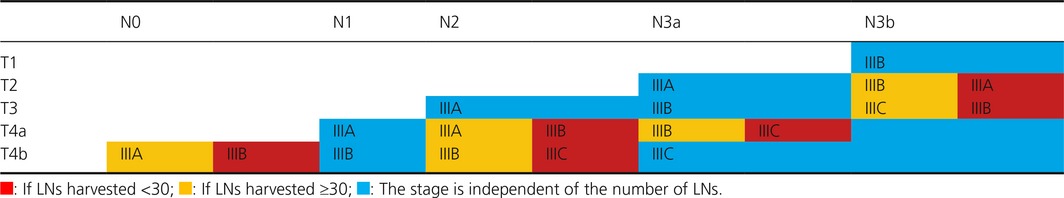
The proposal edition for stage III gastric cancer classification

**Table 3:** Comparison of the performance of the 7th, the 8th edition, and the proposed TNM staging system

	Concordance indices		AIC	Likelihood ratio *χ* ^2^	Linear trend *χ* ^2^
	C‐index	Bootstrap 95% CI			
7th AJCC system	0.624	0.605–0.643	1651.95	6727.243	5917.383
8th AJCC system	0.614	0.596–0.633	1654.69	6725.003	5886.391
Proposed system	0.646	0.628–0.665	1647.30	6732.622	5955.628

## Discussion

Gastric cancer (GC) is the second leading cause of cancer‐related death, with the highest mortality rates found in East Asia 10. Surgical resection remains the main form of treatment. However, despite advances made in treatment strategies over past decades, the prognosis for stage III gastric cancer is still poor. In China, where gastric cancer is endemic, the majority of patients are diagnosed at middle or late stages and have poor overall survival rates 11. Therefore, we focused on stage III, which represents approximately 50% of all entire gastric cancer cases diagnosed in China.

The relationship between retrieved LNs and prognosis is a long‐debated issue, and the number of LNs retrieved from gastrectomy varies widely between Western and Eastern populations 12. There is overwhelming evidence that nodal statuses are strongly influenced by the number of lymph nodes (LNs) resected and that the N stage category increases proportionally to the number of LNs examined 13. If an inadequate number of lymph nodes are examined, the understaging of patients would manifest with the N stage classification 14. Despite several proposals made to do so, the current AJCC staging system still establishes no strict minimum number of resected LNs for adequate staging. However, N3b cannot be assigned when fewer than 16 lymph nodes are harvested. Therefore, the TNM staging system recommends that no <15 LNs be resected for radical gastrectomy. Kong et al. 15 performed a retrospective survival analysis of 8949 GC patients who had undergone curative surgery, and the authors suggested that because the proportion of advanced pN stage cases substantial increases as the number of LNs increases, the minimum number of 15 LNs required for “accurate staging” is not sufficient for accurate staging. Zheng et al. 16 demonstrated that the optimal number of LNs resected is 22 for stage N3a and N3b patients. In this study, we hypothesized that more LNs should be removed in stage III gastric cancer patients than in stages I or II gastric cancer patients. We, therefore, applied cutoff points of LNs harvested (30 produced through X‐tile) exhibiting the highest degree of discriminatory ability and predictive accuracy.

Several important changes were made from the 7th edition to the recently modified 8th edition of the TNM staging system of gastric cancers released in 2016. Changes made to the TNM classification and AJCC tumor stages were based on survival analyses performed for gastric cancer included in the NCDB (U.S.) and Shizuoka Cancer Center (Japan) dataset 17. In this paper, we mainly discuss changes made to the pTNM classification. The introduction of several new subgroups and substages resulted in the creation of a complex and confusing classification for daily clinical use (Fig. 1 and Table S1).

The main change made to the N category in the 8th edition involved splitting the N3 stage into N3a (7–15 positive LNs) and N3b (more than 15 LNs nodes). Although the 7th edition N3 classification was subclassified as N3a and N3b, each subgroup was not an individual determinant of the final TNM stage, which may cause serious problems in underestimating GC severity levels. Although still being controversial 18, there is now more and more proof of the limitations of the 7th AJCC N3 classification, and the need for N classification modifications was raised by various investigators prior to the introduction of the 8th edition AJCC TNM classification 19, 20, 21, 22, 23. Therefore, the 8th edition of the TNM classification adopted numeric classifications for N3, and it was divided into two subgroups in the final TNM stage. The involvement of ≥16 lymph nodes (N3b) was associated with worse outcomes than cases involving 7–15 positive nodes (N3a) according to a series from Italy 19, and similar results were also found in two large Korean studies 20, 24. Our data also confirm that N3a and N3b may represent diseases of differing severity, and the 5‐year survival rate of patients according to the 8th edition N3a classification is also significantly better than that of patients with N3b stage tumors (40.1% vs. 24.7%; *P* < 0.001). Therefore, it appears reasonable to revise the 7th edition pN3(a/b) to a different pN classification even if an analysis of the T1N3b and T2N3b categories was not possible due to an insufficient number of patients.

The N3 stage of the 7th edition system was subdivided into N3a and N3b subcategories in the 8th edition system, directly resulting in the creation of four additional substages in the 8th edition (T1N3b, T2N3b, T3N3b, and T4aN3b). Our data support a change in the former T3/4aN3M0 stages to T3/4aN3aM0 and T3/4aN3bM0 when the LNs removed are ≥30. More importantly, however, we did not find an improvement in the differentiation of survival rates using the newly introduced complex subgroupings of patients with <30 LNs harvested. In our database, only one case involved changing from T2N3M0 (IIIA stage, 7th edition) to T2N3bM0 (IIIB stage, 8th edition), and we excluded this patient from the survival analysis because too few patients had enrolled.

Next, we compared the IIIA, IIIB, and IIIC tumor staging of both editions. T4aN2 and T4bN0 are now classified as stage IIIA, and T4bN2 is now classified as stage IIIB. In our series, partial cases of stage IIIB (T4aN2 and T4bN0) and IIIC (T4bN2) diseases in the 7th edition system were downstaged to IIIA and IIIB in the 8th edition AJCC. Overall, down staging was observed in 36.8% of stage III cases, whereas 6.1% of stage III cases were upstaged. However, when Marrelli et al. 14 compared the 7th system with the 6th edition, they found down staging in 10.4% of cases and upstaging in 27.2% cases. According to our results, substages T4bN0 and T4bN2 showed significantly lower survival rates than IIIA or IIIB, which we attribute to the small number of patients considered (only 10 patients with T4bN0 and 14 patients with T4bN2). Alternatively, T4b may represent diseases of differing severity regardless of whether lymph node metastasis has occurred. Additional studies based on large samples must be conducted for validation purposes.

Staging is a key facet in cancer treatment. The 8th edition of the TNM classification attempts to parse out significant differences in stage III disease survival rates by using a more complex structure compared to that of the 7th edition staging system. However, the 8th TNM edition is not more accurate than the 7th edition in predicting gastric cancer patients’ prognoses. A number of arguments are made. Among our stage III patients, 5‐year survival rates still vary considerably from 55.5% for stage IIIA to 12.0% for stage IIIC. Second, given this context, our results do not support changes in the former T3/4aN3M0 stages to T3/4aN3aM0 and T3/4aN3bM0 when the number of LNs harvested is <30. Finally, for our patient cohort, we found no significant difference between the 8th and 7th AJCC IIIA‐C stage groups when the number of LNs harvested was <30. On the basis of these findings, we propose a new stage grouping approach. The new hybrid TNM classification is based on the number of LNs harvested, on the 7th edition classification and on the 8th edition classification. Using the proposed TNM system, not only the provision of better survival predictions but also the superior categorization of disease severity levels can be achieved through our modified staging approach. In addition, the c‐index value of 0.646 found is higher for the prediction of overall survival rates compared to the TNM classification of the 8th and 7th editions, as shown in Table 3. It is worth noting that this c‐index is lower than those shown in previous studies, which is potentially because stage III GC includes an extremely heterogeneous group of diseases, thus potentially prohibiting the creation of any meaningful stage grouping based solely on local tumor growth and nodal spread patterns. Other variables will likely be shown to significantly influence patient survival rates, such as histological and molecular phenotypes.

Although our results may support the proposed, modified 8th AJCC TNM staging system for GC, this study has several limitations. First, this study was retrospective despite being performed based on a prospectively collected database. It was performed based on data from specialized centers with standardized lymphadenectomy and node retrieval capabilities, and this fact must be considered when comparing results with other cases. Second, we did not analyze the effects of postoperative chemotherapy procedures on prognoses. Third, the cut‐off for LNs harvested through this study (30) was determined using a cohort of patients with a median of 32 examined nodes. These cutoffs may not be optimal values for cohorts of patients with far fewer examined nodes. Fourth, the validation of this proposed classification system in another cohort, particularly in a Western population, should be performed. Fifth, we only focused on stages III patients in this study, without analyzing stages I and II. To address these limitations, our results should be validated for different series based on large sample sizes.

In summary, our analysis does not validate the superior prognostic and discriminating value of the 8th edition AJCC system for stage III gastric cancer. The proposed staging system, comprised of a combination of the number of LNs harvested and the 7th and 8th AJCC classifications, may improve predictive capacities for stage III gastric cancer. However, the finding has to be addressed prospectively in future studies.

## Conflict of Interest

All of the authors declare that they have no potential commercial conflicts of interest relevant to this article.

## Supporting information


**Table S1.** Differences in the TNM classification between the seventh, and eighth editions.

**Figure S1.** Division of patients by the cutoff points produced by X‐tile plot.
