# Supplementary material for: Validation of the American Joint Commission on Cancer (8th edition) changes for patients with stage III gastric cancer: survival analysis of a large series from a Specialized Eastern Center
Source: Cancer Med. 2017 Sep 14;6(10):2179–87. doi: 10.1002/cam4.1118 (PMC5633559; doi:10.1002/cam4.1118)
Supplement: Supplementary file 1 — Table S1. Differences in the TNM classification between the seventh, and eighth editions. [file CAM4-6-2179-s001.docx]

Supplementary table 1. Differences in the TNM Classification Between the Seventh, and Eighth Editions

| AJCC seventh edition | N0 | N1(1-2) | | N2(3-6) | | N3(7-) | | Any N, M1 | |
| --- | --- | --- | --- | --- | --- | --- | --- | --- | --- |
| T1 | IA | IB | | IIA | | IIB | | IV | |
| T2 | IB | IIA | | IIB | | IIIA | |  |  |
| T3 | IIA | IIB | | IIIA | | IIIB | |  |  |
| T4a | IIB | IIIA | | IIIB | | IIIC | |  |  |
| T4b | IIIB | IIIB | |  | | | |  |  |
| Any T, M1 |  | | | | | | | | |
|  | | | | | | | | | |
| AJCC eighth edition | N0 | N1(1-2) | N2(3-6) | | N3a(7-15) | | N3b(16-) | | Any N, M1 |
| T1 | IA | IB | IIA | | IIB | | IIIB | | IV |
| T2 | IB | IIA | IIB | | IIIA | |  |  |  |
| T3 | IIA | IIB | IIIA | | IIIB | | IIIC | |  |
| T4a | IIB | IIIA | IIIA | |  |  |  |  |  |
| T4b | IIIA | IIIB | IIIB | |  | |  |  |  |
| Any T, M1 |  | | | | | | | | |
