# Supplementary figures and images for: Validation of the American Joint Commission on Cancer (8th edition) changes for patients with stage III gastric cancer: survival analysis of a large series from a Specialized Eastern Center
Source: Cancer Med. 2017 Sep 14;6(10):2179–87. doi: 10.1002/cam4.1118 (PMC5633559; doi:10.1002/cam4.1118)

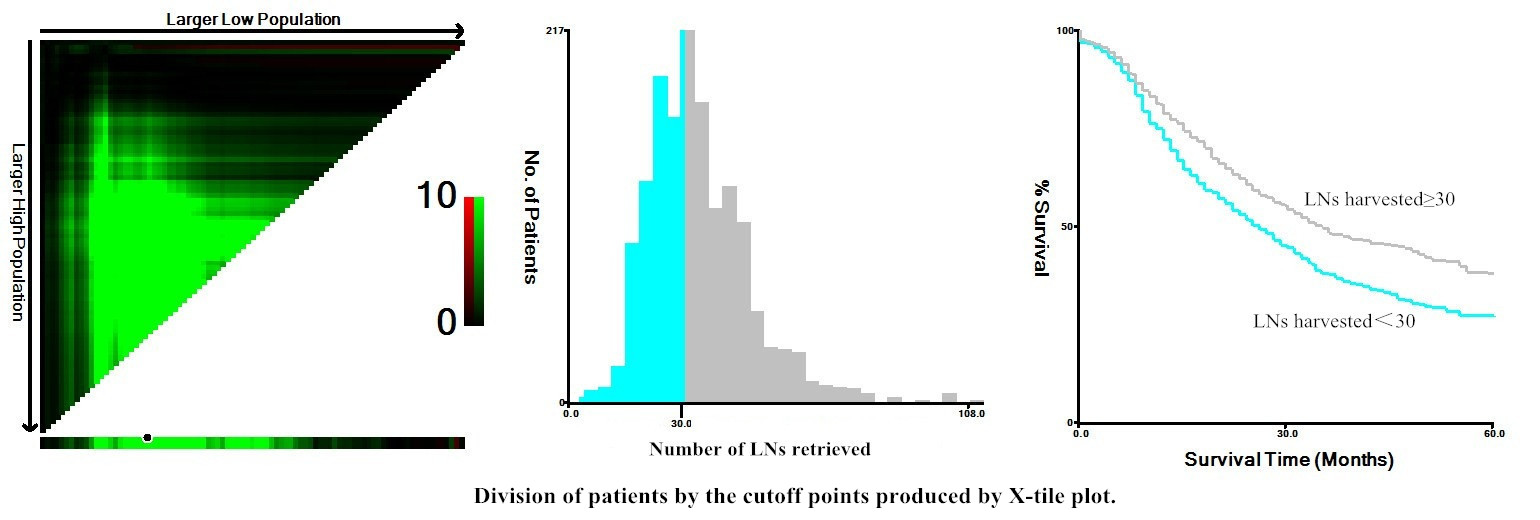

Supplement: Supplementary file 2 — Figure S1. Division of patients by the cutoff points produced by X‐tile plot. [file CAM4-6-2179-s002.tiff]
